# Increased Daytime Sleepiness in Patients with Childhood Craniopharyngioma and Hypothalamic Tumor Involvement: Review of the Literature and Perspectives

**DOI:** 10.1155/2010/519607

**Published:** 2010-12-16

**Authors:** Hermann L. Müller

**Affiliations:** Department of Paediatrics and Paediatric Hematology and Oncology, Klinikum Oldenburg, Rahel-Straus-Straße 10, 26133 Oldenburg, Germany

## Abstract

Childhood craniopharyngiomas are rare embryogenic malformations of the sellar region, presumably derived from Rathke cleft epithelium. The overall survival rates after neurosurgical intervention and/or irradiation are high (92%). However, the quality of survival is frequently impaired due to endocrine deficiencies, sleep disturbances, daytime sleepiness, and severe obesity caused by hypothalamic lesions. Based on self-assessment using nutritional diaries, caloric intake was similar in patients and BMI-matched controls. Analyses of physical activity by accelerometric measurements showed a markedly lower level of physical activity. Significant daytime sleepiness and disturbances of circadian rhythms have been demonstrated in obese childhood craniopharyngioma patients. Daytime sleepiness and obesity in these patients were both correlated with low nocturnal and early morning melatonin levels. Polysomnographic studies in patients with severe daytime sleepiness revealed sleeping patterns typical for secondary narcolepsy. Reports on a beneficial effect of treatment with central stimulating agents supported the hypothesis that secondary narcolepsy should be considered as a rare cause for severe daytime sleepiness in patients with childhood craniopharyngioma.

Craniopharyngiomas are embryogenic malformations of low histological malignancy (WHO I°), which arise from ectoblastic remnants of Rathke's pouch and can be found anywhere along the path of development of Rathke's pouch in hypothalamic and pituitary regions, both of importance in endocrine regulation and satiety modulation [1]. Craniopharyngiomas are the most common intracranial tumors of nonglial origin in the pediatric population, constituting between 1.2 to 4% of all brain tumors and 6 to 9% of pediatric brain tumors. Overall there are 0.5 to 2 new cases per million population occurring each year, 30 to 50% of which are children and adolescents [2]. The peak incidence is at age 5 to 10 years, but they can occur at any age including infancy and pre- and neonatal period [3]. 

Whereas the childhood form of craniopharyngioma mainly presents with an adamantinous histology, the adult type of craniopharyngioma occurs at a peak age of 50–75 years and presents mainly with papillary histology. Other tumors with similar localization but different characteristics on magnetic resonance imaging (MRI) are germinoma, germ cell tumours, langerhans cell histiocytosis, and pilocytic astrocytoma [6]. 

Headaches, visual disturbances, polyuria, reduced growth rates, and weight gain are frequently the first symptoms in the history of patients with childhood craniopharyngioma [7, 8]. The clinical features at the time of diagnosis of craniopharyngioma during childhood are usually unspecific signs of increased intracranial pressure. Major symptoms are headaches, impaired vision (62–84%, primarily in adults), and endocrine failures (52–87%, primarily in children). Chiasmatic involvement may be attended by defects of vision and visual fields. Endocrine deficiencies affect the hypothalamic-pituitary axes for growth hormone (75%), gonadotrophins (40%), ACTH (25%), and thyroid-stimulating hormone (TSH) (25%). 17% of the children with craniopharyngioma present with diabetes insipidus prior to surgery [9]. 

The therapeutic goal is first to relieve symptoms by urgent surgical decompression and second to achieve an early long-term cure by complete resection but without causing further damage to the hypothalamus or optic tract. Postoperative sequelae are deemed unacceptable in patients with preoperatively intact function. However, the optimal primary therapeutic strategy to achieve the correct balance between late sequelae and successful cure remains unknown. Even after complete surgical resection, craniopharyngioma relapses occur in up to 17% of patients [10]. With radical resection, the risk of hypothalamic damage is considerable, especially in craniopharyngioma with suprasellar extension to the hypothalamic area. The appropriate time point of irradiation in patients with residual tumor after incomplete resection is controversial as well [10–13]. 

Although the tumor itself is low-grade histological malignancy and the overall survival rate (92%) of patients is high [9], there is considerable morbidity even when the tumor can be completely resected [5, 7, 9, 10, 14–24]. Childhood craniopharyngioma patients often suffer sequelae of severe obesity. Increased body weight in patients at risk for the development of severe obesity during followup is already detectable at the time of diagnosis of craniopharyngioma [15]. Patients who developed severe obesity presented with a higher body mass index SDS [4] already at the time of diagnosis when compared with patients who kept their normal weight. The evaluation of the patients' history [7] and anthropometric data collected before diagnosis [15] confirmed the observation, that pathogenic mechanisms for the development of later obesity have significant impact on weight already at the time of diagnosis before initiation of therapy. Hypothalamic involvement of craniopharyngioma is the most important risk factor for the development of obesity before and after tumor diagnosis (Figure 1) [9, 10, 15–17, 19, 25]. 

In spite of hormonal substitution, the management of hypothalamic injury-induced hyperphagia is difficult and severe obesity occurs postoperatively in up to 52% of patients with at least one half of these patients having extreme difficulty controlling their desire to eat [9, 24]. Severe obesity has major negative impact on quality of life in survivors of childhood craniopharyngioma [8, 10, 14, 16, 17, 19, 24]. Conventional strategies for weight control are less efficient because of impaired physical activity due to attendant neurological and visual deficits and the complaint of increased daytime sleepiness. 

A German multicenter study on childhood craniopharyngioma patients suggested a secondary hypothalamic disorder as pathogenic factor in patients at risk for severe obesity and increased daytime sleepiness [9]. Müller et al. surveyed a large group of patients with childhood craniopharyngioma and hypothalamic astrocytoma for daytime sleepiness using the German version of the Epworth Sleepiness Scale (ESS) [26]. About 1/3 of the patients reported increased daytime sleepiness, characterized by an ESS score above 10. The severity of their daytime sleepiness was unexpectedly high, especially in obese patients with a BMI > 4 SD [4]. 

Sleep regulation and circadian rhythms are at least partially mediated by hypothalamic structures, for example, the suprachiasmatic nucleus, regulating melatonin secretion [27]. The secretion of melatonin, a pineal indoleamine, occurs during hours of darkness and as it affects sleep patterns it has been tried in treating jet lag and other disorders from delay of sleep because of its possible role in influencing circadian rhythm. Because a destruction or dysfunction of the suprachiasmatic nucleus seems likely in many craniopharyngioma patients with suprasellar tumor extension [28], Müller et al. compared melatonin secretion in severely obese, obese, and in nonobese craniopharyngioma patients [5]. To analyze the influence of obesity and hypothalamic lesions on melatonin secretion, patients with hypothalamic tumors (pilocytic astrocytomas) and obese and normal weight control subjects were also analyzed. The authors compared salivary melatonin concentrations at morning, midday, evening, and nighttime among severely obese, obese, and nonobese patients and normal controls. Salivary melatonin concentrations correlate with melatonin concentrations in plasma [29, 30].

Whereas several studies [31–33] on different patient cohorts have found no significant relation between melatonin secretion and obesity, Birketvedt et al. [34] reported on a rare night eating syndrome characterized by frequent awakening at night, higher nocturnal energy intake, and attenuation of nocturnal rise in plasma melatonin. As hypothesized based on hypothalamic disorders in the severely obese craniopharyngioma patients, decreased melatonin concentrations at nighttime were detected in patients analyzed by Müller et al. [5] (Figures 2(a), 2(b)). The authors speculated that the diurnal rhythm of melatonin was suppressed in obese patients with hypothalamic tumors as craniopharyngioma or pilocytic astrocytoma. As cortisol may also influence wakefulness, salivary cortisol concentrations were compared in all groups to exclude confounding effects. No differences for cortisol serum concentrations were found among the groups. The significant negative correlations between salivary melatonin concentrations in the morning and at nighttime and the ESS scores indicate that reduced nocturnal melatonin secretion may lead to increased daytime sleepiness in patients with childhood craniopharyngioma. The findings suggested that increased daytime sleepiness in patients with childhood craniopharyngioma was associated with decreased nocturnal melatonin levels, which were related to the degree of obesity and the tumor diagnosis. First promising experiences on experimental substitution of melatonin in obese patients with craniopharyngioma supported the hypothesis that increased daytime sleepiness is associated with reduced nocturnal melatonin secretion [20].

The observations confirmed previous reports on agedependency of melatonin secretion [35]. However, in spite of the fact that age-dependent effects were found similarly in all analyzed subgroups and the agedependency had no statistical impact on reported differences in terms of craniopharyngioma-associated melatonin depression, it has to be stated that the preliminary results have to be confirmed by prospective analysis of larger cohorts with more homogeneous agedistribution. Further studies on the hypothesis are part of the German prospective multicenter study KRANIOPHARYNGEOM 2007 on patients with childhood craniopharyngioma [10, 36]. As it has been reported [9] that hypothalamic damage is a risk factor for severe obesity in craniopharyngioma patients, it can be speculated that hypothalamic damage could have been responsible for disturbances in melatonin secretion. This speculation is supported by similar findings for patients with hypothalamic tumors of other histology such as pilocytic astrocytoma [5].

Studies on physical activity using accelerometric analysis of movement counts revealed that physical activity was reduced in the group of craniopharyngioma patients with obesity and hypothalamic involvement when compared with age and BMI-matched controls [25]. Caloric intake was similar in normal controls (1027 healthy nonobese subjects, representative sample of the 7 to 16 year-old German population with an age distribution: 11.3 ± 2.7 years) and craniopharyngioma patients (27 patients, age distribution: 11.7 ± 2.6 years) and had no significant impact on the degree of obesity and the physical activity in analyzed cohorts [25]. Hypothalamic involvement of craniopharyngioma had major negative impact on functional capacity and quality of life and was a major risk factor for the development of severe obesity in survivors of childhood craniopharyngioma [5, 7–10, 14–21]. 

Reports [5, 22, 25] on increased daytime sleepiness and reduced physical activity in patients with craniopharyngioma support the hypothesis that physical activity might be decreased in these patients due to yet unknown neuroendocrine disorders. On the other hand, sleep at night was severely disturbed in many patients with increased daytime sleepiness [5]. Accordingly, Müller et al. analyzed daytime sleepiness and polysomnographic patterns in patients with childhood craniopharyngioma in order to define further risk factors for severe obesity.

Since sleep regulation and circadian rhythms are at least partially mediated by hypothalamic structures, for example, the suprachiasmatic nucleus, Müller et al. conducted a two-night polysomnography (PSG) and a multiple sleep latency test (MSLT) consisting of four or five 20-minute naps with nine obese craniopharyngioma patients and one patient with an astrocytoma of the pituitary stalk displaying acute daytime sleepiness [20]. The MSLT was developed to render a better diagnostic sensitivity and specificity in the diagnosis, and usually two or more SOREMP in the MSLT are regarded as necessary for the diagnosis. Usually a mean sleep latency of <5 minutes should be observed for the diagnosis of narcolepsy. The diagnostic validity of MSLT in early infancy is controversial. However, the youngest patient included in our MSLT analyses was 10 years of age [20]. Only two patients showed an obstructive sleep apnea syndrome (OSAS), the usual sleep-related disorder in acutely obese patients. However, seven patients fulfilled the classic PSG criteria for secondary narcolepsy or hypersomnia. These results were unexpected since none of the patients complained of cataplexy, hypnagogic hallucinations, or sleep paralysis on inquiry. What is particularly noteworthy is that recent research has suggested a hypothalamic disorder in narcolepsy. A defect in the orexin II receptor is responsible for canine narcolepsy [37] and orexin knockout mice show characteristic features of narcolepsy [38]. Orexin is expressed exclusively in the lateral hypothalamus, and the orexin receptors seem to be wider spread [39]. In human narcoleptics, 8 of 10 had orexin A below the detection limit of the assay used [40]. Despite excessive research in this field, only one patient could be identified with a genetic defect in the orexin system [41]. In autopsy of narcoleptic patients, the lack of orexin neurons in the lateral hypothalamus was observed in 10 cases [41, 42]. 

It has also been reported that systemic administration of orexin relieves narcoleptic symptoms in dogs [43, 44]. The peculiar finding that sleep and sleep attacks in narcoleptic patients are initiated by a sleep onset REM period (SOREMP) was recognized in the early 1960s [45]. Since then, this PSG finding is regarded as a phenomenon occurring almost exclusive in narcolepsy, although there are some descriptions of SOREM in subjects without narcolepsy. García-Borreguero et al. [46] reported that glucocorticoid replacement therapy in Addison's patients was permissive for decreased REM latency when hydrocortisone was taken at bedtime. MSLT was not performed in this study. All patients with SOREM were under treatment with hydrocortisone replacement therapy in the study of Müller et al. [20]. However, hydrocortisone replacement treatment alone cannot explain the excessive daytime sleepiness in analyzed patients as this is standard treatment for craniopharyngioma patients, including those not suffering severe daytime sleepiness.

Secondary narcolepsy is a rare disorder. However, several case reports were published on secondary narcolepsy, mainly reporting on patients with tumorous conditions in the hypothalamic area [47, 48]. Diagnostic criteria vary, but all patients presented with hypersomnia as a leading pathology. Interestingly, the majority of reported patients show hypersomnia, but not cataplexy, hallucinations, or sleep paralysis. In fact, a medline search yielded over 30 cases of secondary narcolepsy without cataplexy during the last 50 years, but yielded only 13 cases with secondary cataplexy. These cases are surprisingly very heterogenic and only two cases had tumors in the area of the hypothalamus [49], two cases had tumors in the brain stem pontomedullary astrocytoma [50], glioblastoma of rostral brain stem [51], one patient had a frontal lobe tumor [52], five patients had meningioma [53], and five patients had meningeal carcinomatosis [54]. 

Not all patients with a tumorous condition in the hypothalamic area suffer from hypersomnia, and even less from cataplexy. This is surprising, since deficiency of orexin is regarded as the cause of hypersomnia and cataplexy in idiopathic narcolepsy. Cases with secondary cataplexy in the literature seem to have more widespread tumor disease than cases with secondary hypersomnia. This leads to speculation that there must be some other pathology operating in addition to orexin deficiency to produce cataplexy in idiopathic narcolepsy. This hypothesis is supported by the fact that some patients with clear idiopathic narcolepsy and cataplexy have normal orexin levels in cerebrospinal fluid [40].

In concert with findings [5, 20] suggesting that increased daytime sleepiness is a common complaint in patients with childhood craniopharyngioma and that the incidence seems to be equal in obese and normal weight patients, reported results [22] together with current research on narcolepsy lead to the conclusion that secondary hypersomnia and secondary narcolepsy may be contributing causes for increased daytime sleepiness and weight control difficulties in obese craniopharyngioma patients. Preliminary positive experiences with central stimulating agent treatment (Modafinil or Methylphenidate) in patients with childhood craniopharyngioma and secondary narcolepsy support this speculation [22, 55]. 

Based on the literature [56, 57], radical surgery with potential damage to hypothalamic structures and consecutive increased daytime sleepiness is no appropriate treatment strategy in patients with hypothalamic involvement of childhood craniopharyngioma. For such patients innovative treatment strategies are warranted after incomplete resection. Accordingly, in KRANIOPHARYNGEOM 2007 quality of life, event-free survival and overall survival rates in patients (age ≥5 years at diagnosis and at incomplete resection) are currently analyzed after randomization of the time point of irradiation after incomplete resection (immediate irradiation *versus* irradiation at progression of residual tumor). The schedule of prospective data collection and the set and definition of parameters is based on a European consensus [13]. Standardized European data sets on a rare disease such as childhood craniopharyngioma should help to increase cohort sizes and facilitate common data evaluation [10]. Hopefully, this international study will lead to treatment recommendations that prevent severe sequelae such as increased daytime sleepiness and secondary narcolepsy in patients with childhood craniopharyngioma.

## Figures and Tables

**Figure 1:**
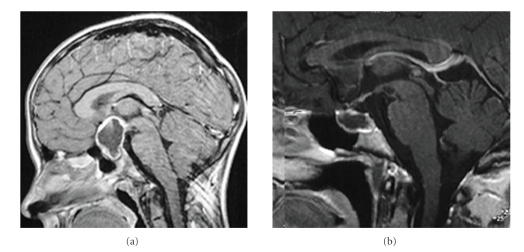
Obesity and daytime sleepiness in relation to the localization of craniopharyngioma. The patient whose preoperative MRI (a) showed a large tumor extending to the suprasellar region and infiltrating the hypothalamus developed severe daytime sleepiness and, consequently, obesity (BMI: +14 SD [4]). The patient with a childhood craniopharyngioma of intrasellar localization seen in Figure 1(b) maintained normal weight (BMI: +1 SD [4]) and developed no daytime sleepiness.

**Figure 2:**
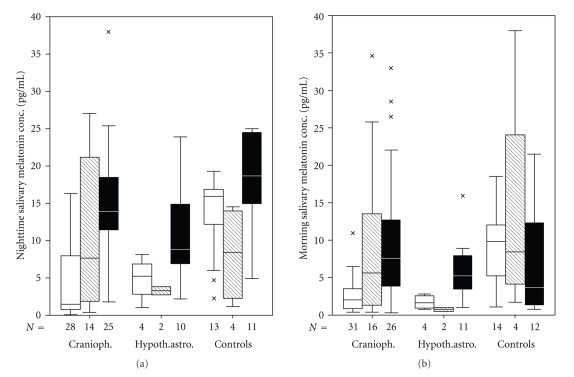
Salivary melatonin concentrations at nighttime (a) and in the morning (b) in patients with childhood craniopharyngioma, hypothalamic pilocytic astrocytoma, and controls in relation to the degree of obesity (body mass index [BMI] <2 SD [filled black boxes], BMI 2–4 SD [hatched gray boxes], or BMI ≥ 4 SD [open boxes]). The horizontal line in the middle of the box depicts the median. Edges of the box mark the 25th and 75th percentile. Whiskers indicate the range of values that fall within 1.5 boxlengths. Values more than 1.5 box-length from the 25th and 75th percentiles are marked by an asterix. (Modified from [5], with the kind permission of Endocrine Press.)
